# Editorial

**Published:** 1849-07

**Authors:** 


### THE MEDICAL EXAMINER.

### PHILADELPHIA, JULY, 1849.

Dr. David H. Tucker, one of the Editors of the Medical Examiner, has been appointed Professor of the Theory and Practice of Medicine in Hampden Sidney College, Richmond, Virginia.

---

### STANDING COMMITTEES OF THE AMERICAN MEDICAL ASSOCIATION, FOR 1849–50.

As some errors were accidentally made in the list published in the June number of the Medical Examiner, we republish below a corrected one, furnished through the kindness of Dr. Stillé, one of the Secretaries.

*Committee on Medical Science.*

Dr. Usher Parsons, Providence, R. I., Chairman.

| | |
|---|---|
| Dr. J. Bigelow, Boston. | Dr. Jas. Moultrie, Charleston, S. C. |
| " J. B. S. Jackson, Boston. | " G. Emerson, Philadelphia. |
| " A. B. Malcolm, Dubuque, Iowa. | " D. King, Newport, R. I. |

*Committee on Practical Medicine.*

Dr. J. K. Mitchell, Philadelphia, Chairman.

| | |
|---|---|
| Dr. R. La Roche, Philadelphia. | Dr. James Jones, New Orleans. |
| " F. West, Philadelphia. | " R. D. Arnold, Savannah. |
| " J. A. Swett, New York. | " —— Smith, Indiana. |

*Committee on Surgery.*

Dr. R. D. Mussey, Cincinnati, Chairman.

| | |
|---|---|
| Dr. W. M. Awl, Columbus, Ohio. | Dr. L. A. Dugas, Augusta, Ga. |
| " A. B. Shipman, Syracuse, N. Y. | " S. Parkman, Boston. |
| " G. Fox, Philadelphia. | " J. R. Wood, New York. |

*Committee on Obstetrics.*

Dr. T. G. Prioleau, Charleston, S. C., Chairman.

| | |
|---|---|
| Dr. L. D. Ford, Augusta, Ga. | Dr. H. F. Askew, Wilmington, Del. |
| " Robert Lebby, Charleston, S. C. | " John Evans, Chicago, Ill. |
| " Josiah Bartlett, Stratham, N. H. | " Isaac Lincoln, Brunswick, Me. |

*Committee on Medical Education.*

Dr. J. Roby, Baltimore, Md., Chairman.

| | |
|---|---|
| Dr. Blatchford, Troy, N. Y. | Dr. F. A. Ramsey, Knoxville, Tenn. |
| " G. C. M. Roberts, Baltimore. | " Geo. Sumner, Hartford, Conn. |
| " R. W. Silvester, Norfolk, Va. | " W. H. Rockwell, Brattleboro, Vt. |

*Committee on Medical Literature.*

Dr. Alfred Stillé, Philadelphia, Chairman.

| | |
|---|---|
| Dr. F. G. Smith, Philadelphia. | Dr. A. T. Morris, Montgomery, Ala. |
| " T. H. Yardley, Philadelphia. | " J. Fithian, Woodbury, N. J. |
| " P. C. Gaillard, Charleston, S. C. | " J. B. Johnson, St. Louis, Mo. |

*Committee on Publication.*

Dr. I. Hays, Philadelphia, Chairman.

| | |
|---|---|
| Dr. A. Stillé, Philadelphia. | Dr. B. F. Barker, Norwich, Conn. |
| " H. J. Bowditch, Boston. | " Isaac Wood, New York. |
| " D. F. Condie, Philadelphia. | " N. J. Pittman, Rocky Mt., N. C. |

*Committee on Forensic Medicine.*

Dr. A. H. Stevens, N. Y., Chairman.

| | |
|---|---|
| Dr. Luther V. Bell, Boston. | Dr. Robert Watts, New York. |
| " Pliny Earle, New York. | " R. S. Steuart, Baltimore. |
| " W. H. Rockwell, Vt. | " J. Knight, New Haven, Conn. |

*Committee on Indigenous Botany and Materia Medica.*

Dr. Eli Ives, New Haven, Chairman.

| | |
|---|---|
| Dr. G. L. Corbyn, Warwick Co. Va. | Dr. B. B. Lenoir, Roane Co. Tenn. |
| " H. R. Frost, Charleston, S. C. | " W. B. Cochran, Middleburg, Va. |
| " W. H. Davis, Baltimore. | " J. P. Harrison, Cincinnati. |

*Committee on Hygiene.*

Dr. J. M. Smith, N. Y., Chairman.

| | |
|---|---|
| Dr. A. K. Gardner, New York. | Dr. R. S. Holmes, St. Louis, Mo. |
| " E. Jarvis, Dorchester, Mass. | " G. Emerson, Philadelphia. |
| " A. T. M. Cooke, Norfolk, Va. | " J. C. Simonds, New Orleans. |

---

ST. LOUIS MEDICAL AND SURGICAL JOURNAL.

We cheerfully give place to the following letter from one of the Editors of the above Journal, and at the same time express our sympathy with them in their loss, and our hopes that the suspension may be but temporary. The St. Louis Journal is one of our exchanges whose appearance we always anticipate with pleasure.

St. Louis, May 21st, 1849.

(To the Editors of the Western Lancet.)

*Gentlemen,*—Will you be good enough to state, in the next number of your Journal, that during the late destructive fire, which well nigh consumed our entire city, the office at which the St. Louis Medical and Surgical Journal was printed was destroyed, and with it all the type, manuscript, back numbers, and other material of the Journal. The next number was to have been issued in a few days. At present everything is in confusion; but as soon as we have an opportunity of looking around, other arrangements will be made, and the work commenced again with as little delay as possible; in the mean time we take this method of informing our readers of the cause of our suspension, and to bespeak their kindly indulgence. We would also be gratified if other Journals would make the same announcement.

With sentiments of respect and esteem,

I am, gentlemen, your obedient servant,

W. McPheeters.

THE CHOLERA IN PHILADELPHIA.

The following table shows the whole number of cases of cholera, and deaths from the same disease, from the 30th of May, when it was first officially reported, up to 27th June, inclusively:

| | Cases. | Deaths. | | Cases. | Deaths. |
|---|---|---|---|---|---|
| May 30, | 3 | 3 | June 18, | 3 | 1 |
| June 1, | 2 | 0 | " 19, | 6 | 2 |
| " 2, | 1 | 0 | " 20, | 3 | 2 |
| " 5, | 1 | 0 | " 21, | 10 | 4 |
| " 7, | 2 | 2 | " 22, | 5 | 1 |
| " 12, | 2 | 1 | " 23, | 5 | 2 |
| " 13, | 3 | 1 | " 24, | 3 | 3 |
| " 14, | | 1 | " 25, | 20 | 8 |
| " 15, | 2 | 0 | " 26, | 21 | 10 |
| " 16, | 2 | 2 | " 27, | 43 | 12 |
| " 17, | 2 | 1 | | | |

By the above table it will be seen that during a period of 29 days, there has been an average of five cases and a fraction over one death per diem. These facts show that although the disease is in our midst, and the number of cases has increased within the two days previous to our going to press, it has not as yet assumed a violent form, notwithstanding several days of the most trying hot weather. Our city authorities are fully awake to the necessity of prompt action, and have already established dispensaries in various parts of the city and districts, and are expected shortly to open hospitals for the reception of the sick poor. By these wise sanatory measures, it is hoped that the epidemic will be robbed of much of its terrors.

---

HEALTH OF THE CITY.

By the bill of mortality for the week ending June 23, 1849, it will be seen that the whole number of interments in the City and Liberties was 184. This is a considerable increase over the week previous, but for the same time last year 244 interments were reported by the Board of Health. The weather then was not as hot, and there was no Asiatic cholera, or any other epidemic prevailing.

HEALTH OFFICE, June 23, 1849.

*Interments in the City and Liberties of Philadelphia, from the 16th to the 23d of June.*

Abscess of Hip-Joint, 1; Apoplexy, 3; Cancer, 1; do. of the Uterus, 1; Caries of Spine, 1; Casualties, 6; Croup, 3; Congestion of the Lungs, 1; do. of the Brain, 5; Cholera Asphyxia, 14; do. Infantum, 20; do. Morbus, 5; Consumption of the Lungs, 14; Convulsions, 11; Diarrhœa, 2; Dropsy, 3; do. of the Spine, 1; do. of the Head, 3; do. in the Breast, 1; Disease of the Brain, 4; do. of the Heart, 1; Drowned, 7; Dysentery, 3; Debility, 4; Enlargement of the Heart, 1; Fever, Bilious, 1; do. Congestive, 1; do. Nervous, 1; do.

Scarlet, 4; Typhoid, 3; Gangrene, 1; do. of Foot, 1; Hemorrhage, 1; Inflammation of the Brain, 3; do. of the Bronchi, 5; do. of the Lungs, 4; do. of the Stomach and Bowels, 3; do. of the Uterus, 1; do. of the Peritonæum, 2; Mania a Potu, 4; Marasmus, 3; Measles, 1; Old Age, 5; Palsy, 2; Scirrhus of Stomach, 1; do. of Eye Ball, 1; Small Pox, 4; Still Born, 7; Unknown, 8; Whooping Cough, 1—93 Adults, 91 Children.

Of the above there were—Under one year 48; from 1 to 2, 16; 2 to 5, 11; 5 to 10, 3; 10 to 15, 6; 15 to 20, 7; 20 to 30, 18; 30 to 40, 32; 40 to 50, 17; 50 to 60, 6; 60 to 70, 10; 70 to 80, 8; 80 to 90, 2.—Total, 184.

From the Almshouse, 8; People of Color, 20; from the Country, 0. By order of the Board of Health.

